# Combined breast conservation therapy versus mastectomy for BRCA mutation carriers – A systematic review and meta-analysis

**DOI:** 10.1016/j.breast.2021.02.001

**Published:** 2021-02-04

**Authors:** M.G. Davey, C.M. Davey, É.J. Ryan, A.J. Lowery, M.J. Kerin

**Affiliations:** a The Lambe Institute for Translational Research, National University of Ireland, Galway, Ireland; b Department of Surgery, Galway University Hospitals, Galway, Ireland; c School of Medicine, National University of Ireland, Galway, Ireland; d Royal College of Surgeons in Ireland, Dublin, Ireland

**Keywords:** Breast cancer, Genetics, Surgical oncology, BRCA mutations

## Abstract

**Background:**

The non-inferiority of combined breast conservation surgery (BCS) and radiotherapy (breast conservation therapy or BCT) compared to mastectomy in sporadic breast cancer cases is well recognised. Uncertainty remains regarding optimal surgical practice in BRCA mutation carriers.

**Aims:**

To evaluate the oncological safety of combined BCT versus mastectomy in BRCA mutation carriers following breast cancer diagnosis.

**Methods:**

A systematic review was performed as per PRISMA and MOOSE guidelines. Observational studies comparing BCS and mastectomy in BRCA carriers were identified. Dichotomous variables were pooled as odds ratios (OR) using the Mantel–Haenszel method. Log hazard ratios (lnHR) for locoregional recurrence (LRR), contralateral breast cancer, disease-free and overall survival and their standard errors were calculated from Kaplan-Meier or cox-regression analyses and pooled using the inverse variance method.

**Results:**

Twenty three studies of 3807 patients met inclusion criteria; 2200 (57.7%) were BRCA1 and 1212 (31.8%) were BRCA2 carriers. Median age at diagnosis was 41 years with 96 months follow up. BCS was performed on 2157 (56.7%) while 1408 (41.5%) underwent mastectomy. An increased risk of LRR was observed in patients treated with BCS (HR:4.54, 95% Confidence Interval: 2.77–7.42, *P* < 0.001, heterogeneity (*I*^*2*^) = 0%). However, the risks of contralateral breast cancer (HR:1.51, 95%CI: 0.44–5.11, *P* = 0.510, *I*^*2*^ = 80%), disease recurrence (HR:1.16, 95%CI: 0.78–1.72, *P* = 0.470, *I*^*2*^ = 44%), disease-specific recurrence (HR:1.58, 95%CI: 0.79–3.15, *P* = 0.200, *I*^*2*^ = 38%) and death (HR:1.10, 95%CI: 0.72–1.69, *P* = 0.660, *I*^*2*^ = 38%) were equivalent for combined BCT and mastectomy.

**Conclusions:**

Survival outcomes following combined BCT is comparable to mastectomy in BRCA carriers. However, the risk of LRR is increased. Patient counselling should be tailored to incorporate these findings.

## Highlights

• BRCA carriers see similar survival after breast conservation therapy and mastectomy.
• Locoregional recurrence rates are increased following breast conservation.
• Surgical counselling may reflect these findings for BRCA mutation carriers.

## 1 Introduction

In the western world, the lifetime risk of developing sporadic breast cancer is 12.4% [1]. Estimations suggest that 10% of breast cancer is due to hereditary predisposition, with family history amplifying the likelihood of developing the disease [2]. Moreover, this risk is exponentially increased in those harbouring breast cancer gene (BRCA) mutations. These genes are highly penetrant; there is a reported cumulative risk of almost 80% for female breast cancers in BRCA1 mutations, as well as a 50% risk for BRCA2 mutation carriers [[3], [4], [5]].

Surgical resection is the primary curative strategy for breast cancer. In cases of sporadic breast cancers this frequently involves breast conservation therapy (BCT) in the form of combined breast conservation surgery (BCS) and adjuvant radiotherapy, provided clear margins of resection are achieved [6]. Risks of locoregional recurrence (LRR) have been overestimated in the past, and in recent times, the paradigm has shifted such that omitting adjuvant radiotherapy may even be considered in some select patients with breast cancer, given their predicted risk of LRR being low following BCS [7].

However, in patients possessing BRCA-mutations, clinical outcomes such as locoregional control, oncological outcomes, and expected prognoses are less certain [8]; the international perception is that the risk of new primary cancers is so high as to make breast conservation a dangerous therapeutic strategy, and there is a vogue towards bilateral mastectomy being the most appropriate strategy for surgical management of newly diagnosed breast cancer in BRCA mutation carriers [6,9,10]. Currently, there are unanswered questions concerning survival advantage including those related to the underlying breast cancer subtype and prognostic criteria related to the index cancer; (eg. BRCA1 mutation carriers have the tendency to develop triple negative breast cancers) [11].

Surgical strategy also impacts the requirement for second operations, while risk reduction strategies are beneficial for BRCA mutation carriers yet to develop cancers [12]; in those diagnosed with primary cancer, the impact of local field radiotherapy on the index breast may have significant impact on future tumours, and indeed survival. Currently, ethical considerations limit the practicality of performing a prospective randomised-controlled trial on this topic, adding further uncertainty regarding optimal surgical oncological practice for those with BRCA mutations.

Given this uncertainty, the primary aim of the current study was to evaluate the oncological safety of BCT versus mastectomy with respect to LRR, new primary breast cancers, and survival in BRCA mutation carriers.

## 2 Materials and methods

### 2.1 Search strategy

A systematic review was performed in accordance to the Preferred Reporting Items for Systematic Reviews and Meta-Analyses (PRISMA) checklist and Meta-Analyses Of Observational Studies in Epidemiology (MOOSE) guidelines [13,14]. The first and second authors conducted a comprehensive electronic search of the EMBASE, SCOPUS and PUBMED databases for studies to be considered in this analysis, the latest of which occurred on the 30^th^ of November 2020. The search terms “BRCA” AND “lumpectomy” OR “BRCA” AND “breast conserving surgery” OR “BRCA” AND “breast conserving therapy” OR “hereditary breast and ovarian syndrome” AND “lumpectomy” OR “hereditary breast and ovarian syndrome” AND “breast conserving surgery” OR “hereditary breast and ovarian syndrome” AND “breast conserving therapy”. Secondary referencing was conducted by manually reviewing reference lists of potentially eligible studies. Manuscripts published in languages other than English were excluded. Studies were not restricted based on year of publication. All titles were initially screened, and studies deemed appropriate had their abstracts and full texts reviewed. In studies with data derived from the same patient sample, studies providing the most relevant survival data were included for analyses.

### 2.2 Inclusion and exclusion criteria

Studies meeting the following inclusion criteria were considered for inclusion in this analysis [1]: included patients carrying BRCA mutations with histopathological confirmation of breast cancer diagnoses [2], patients underwent surgical resection of their breast carcinoma [3], patient clinicopathological, immunohistochemical, treatment and survival data were available. Studies meeting the following criteria were excluded from this analysis [1]: assessed the oncological safety of prophylactic or bilateral mastectomies only [2], review articles [3], studies including less than 5 patients in their series or case reports [4], editorial articles, and [5] conference abstracts.

### 2.3 Data extraction and quality assessment

A formal literature search was performed by the first and second authors (M.G.D and C.M.D) using a predefined search strategy. Duplicate studies were removed. Both reviewers assessed the retrieved manuscripts for the above inclusion and exclusion criteria, before extracting [1]: First author name [2], year of publication [3], nature of study [4], country [5], number of patients in the series [6], number of BRCA 1 and BRCA 2 mutations carriers in each series [7], surgeries performed [8], clinicopathological data [9], treatment modalities and characteristics, and [10] survival data. Data specific to clinicopathological and treatment characteristics (expressed as risk ratios (RR), 95% confidence intervals (95% CI) and *P*-values) and clinical outcomes and survival (expressed as hazards ratios (HR), 95% CI, and *P*-values) were directly extracted from study manuscripts. Risk of bias and methodology quality assessment was conducted in concordance to the Newcastle-Ottawa scale [15]. In case of discrepancies in opinion between both reviewers, a third reviewer was asked to arbitrate (É.J.R). Studies reporting data from the same centre were evaluated for the duplication of patients data; studies determined to possess data overlapping with different studies were removed.

## 3 Results

### 3.1 Literature search

**Fig. 1:**
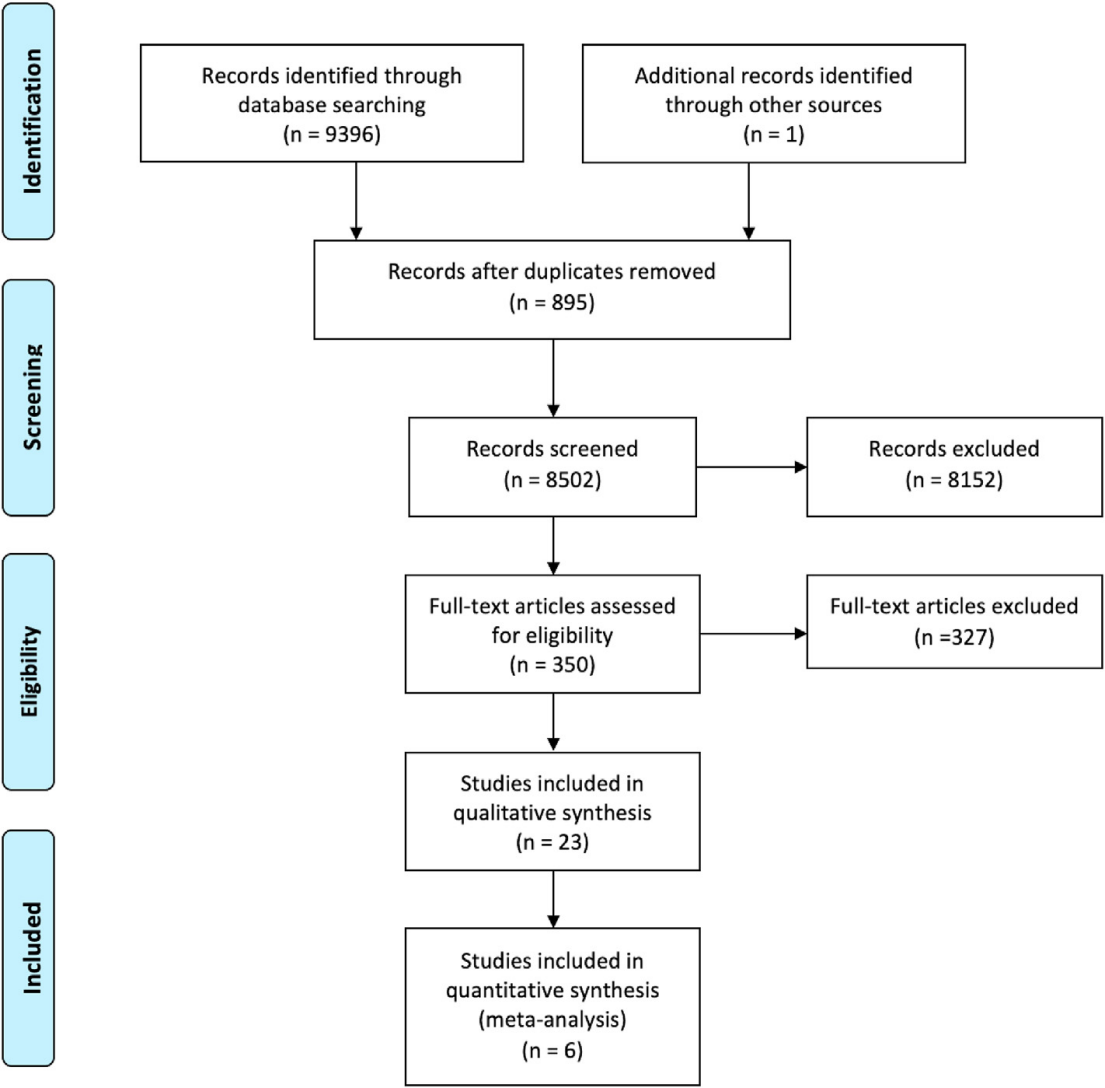
PRISMA flow diagram detailing the systematic search process.

The initial literature search yielded a total of 9397 studies. Following removal of 895 duplicate studies, 8502 studies were screened for relevance for inclusion, of which 350 studies had their abstracts reviewed. Sixty four studies had their full manuscripts screened, before 23 studies were included for meta-analysis (Fig. 1) [[16], [17], [18], [19], [20], [21], [22], [23], [24], [25], [26], [27], [28], [29], [30], [31], [32], [33], [34], [35], [36], [37], [38], [39]]. The included studies are illustrated in Table 1.

**Table 1:** Details regarding the 22 independent patient cohorts included in this systematic review.

Author	Year	Study Type (LOE)	Country	BRCA (N)	BRCA1	BRCA2	BCS (N)	Mastectomy (N)	Median age (years)	Follow up (months)	NOS
Robson	2005	RC (III)	US	87	62	25	87	–	43	76	6
Turner	1999	RC (III)	US	8	5	3	8	–	36	174	5
Garcia-Etienne	2009	RC (III)	Italy	54	26	28	54	–	36	48	6
Yoon	2019	RC (III)	Korea	51	33	18	51	–	44	85	6
Nilsson	2014	RC (III)	Sweden	162	–	–	45	118	43	155	7
Chiba	2016	RC (III)	US	173	100	73	60	109	46	41	6
Haffty	2002	RC (III)	US	22	15	7	22	–	34	168	6
Ye	2020	RC (III)	China	63	–	–	63	–	41	61	5
Van den Broek	2018	RC (III)	Netherlands	261	190	71	125	136	39	40	6
Robson	1995	RC (III)	US	35	27	9	35	–	–	124	5
Drooger	2015	RC (III)	Netherlands	691	517	174	349	337	–	109	7
Huang	2020	RC (III)	China	176	–	–	44	132	–	48	5
Pierce	2010	RC (III)	US	655	394	261	302	353	42	103	6
Kirova	2010	RC (III)	France	27	19	8	–	–	43	167	5
Pierce	2016	RC (III)	US	160	123	37	160	–	40	95	6
Metcalfe	2011	RC (III)	Canada	396	259	142	396	–	42	126	6
Brekelmens	2007	RC (III)	Netherlands	260	90	170	111	135	43	52	6
Eccles	2001	RC (III)	UK	75	75	–	36	39	38	107	7
El Tamer	2004	RC (III)	US	51	30	21	21	30	48	79	6
Robson	1998	RC (III)	US	30	23	7	9	19	36	66	6
Cao	2019	RC (III)	China	103	31	72	103	–	45	80	6
Foulkes	1997	RC (III)	US	12	12	–	11	–	45	37	5
Bernstein-Mohlo	2020	RC (III)	Israel	255	169	86	127	128	44	71	7

LOE; level of evidence, N; number, BCS; breast conservation surgery, NOS; Newcastle-Ottawa Scale, RC; retrospective cohort, US; United States, UK; United Kingdom.

### 3.2 Study characteristics

This review included 3807 patients from 10 countries. Of these, 2200 possessed BRCA1 mutations (57.7%) and 1212 possessed BRCA2 mutations (31.8%). The median age at the time of breast cancer diagnosis was 41 years and median follow up was 96 months. Two thousand and one hundred and fifty seven patients underwent BCS (56.7%), while 1579 underwent mastectomy (41.5%).

### 3.3 Histopathological and immunohistochemical data

Invasive ductal carcinoma histopathological subtype *(P* < 0.001), pathological tumour stages 1–2 *(P* < 0.001) and lymph node negativity *(P* < 0.001) were all associated with undergoing BCS (all Fisher’s exact test, †). Data not associated with surgical approach are demonstrated in Table 2.

**Table 2:** Associations of histopathological and immunohistochemical data and breast conservation and mastectomy.

Parameter	BCT	Mastectomy	*P*-value
T1/2	1589	812	<0.001^b^^a^
T3	12	71	
IDC	1078	230	<0.001^b^^a^
Not IDC	299	186	
Grade 1/2	291	135	0.562^a^
Grade 3	741	320	
LN negative	1206	592	<0.001^b^^a^
LN positive	599	426	
ER positive	655	390	0.141^a^
ER negative	1027	541	
PgR positive	167	122	0.131^a^
PgR negative	359	210	
HER2 positive	32	25	0.750^a^
HER2 negative	524	375	

BCT; breast conservation therapy, T; tumour stage, IDC; invasive ductal carcinoma histological subtype, LN; lymph node, ER; estrogen receptor, PgR; progesterone receptor, HER2; human epidermal growth factor receptor-2.

a Denotes statistical significance.

b Fisher’s exact test.

### 3.4 Treatment characteristics

Neoadjuvant chemotherapy (NAC) prescription was reported in two analyses; 42.6% of patients underwent NAC in the BCS group (203/477) vs. 51.7% in the mastectomy group (240/464) (P = 0.005, †). In the BCS group, 28.5% of patients with ER + disease received adjuvant endocrine therapies (431/1510–12 studies), while 31.4% did so in the mastectomy group (338/1076–5 studies) (*P* = 0.116, †). Of patients undergoing BCS, 68.7% received adjuvant chemotherapy (AC) (885/1289–12 studies) *vs.* 60.7% in those undergoing mastectomy (526/866–4 studies) (*P* < 0.001, †). In the BCS group, 97.3% underwent adjuvant radiotherapy (1337/1374–9 studies), as did 41.8% in the mastectomy group (363/867–5 studies) (*P* < 0.001, †). In those undergoing BCS, 52.7% underwent prophylactic oophorectomy (736/1396–7 studies) *vs.* 50.7% in the mastectomy group (544/1072–5 studies) (*P* = 0.155, †). No included studies outlined data concerning surgical management of the axilla (Table 3).

**Table 3:** Associations of treatment characteristics and breast conservation surgery and mastectomy.

Treatment parameter	BCS	Mastectomy	*P*-value
NAC	203	240	0.005^b^^a^
No NAC	274	224	
EHT	431	338	0.116^a^
No EHT	1079	738	
AC	885	526	<0.001^b^^a^
No AC	404	340	
ALND No ALND	N/R	N/R	N/C
XRT	1337	363	<0.001^b^^a^
No XRT	32	504	
Oophorectomy	763	544	0.155^a^
No oophorectomy	660	528	

BCS; breast conservation surgery, NAC; neoadjuvant chemotherapy, EHT; Endocrine hormonal therapy, AC; Adjuvant systemic chemotherapy, ALND; axillary lymph node dissection, XRT; adjuvant radiotherapy, N/R; not reported, N/C; not calculatable.

a Denotes statistical significance.

b Fisher’s exact test.

### 3.5 Locoregional recurrence

**Fig. 2:**
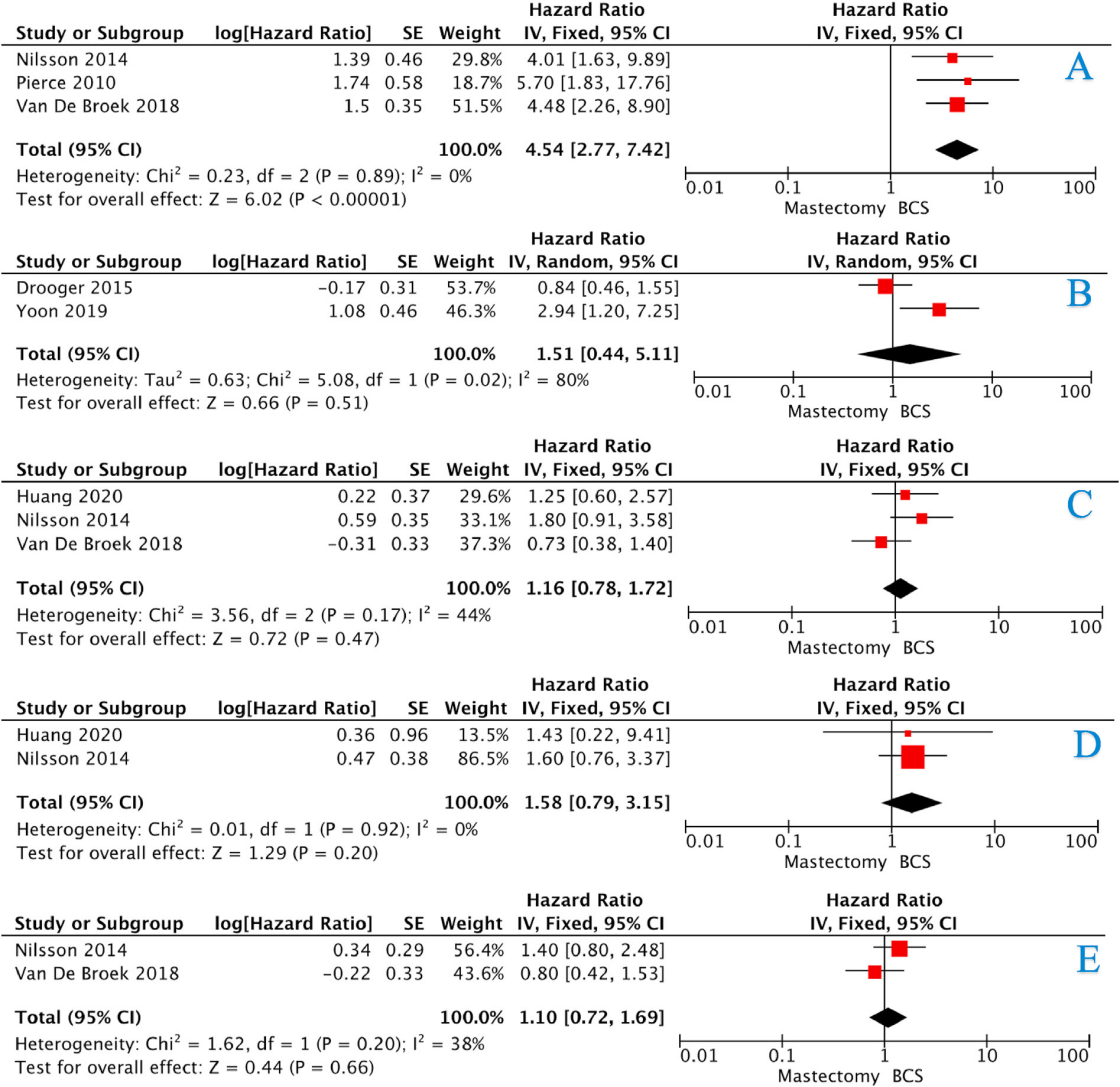
Forest plots illustrating (A) locoregional recurrence, (B) contralateral breast cancer, (C) disease-free survival, (D) disease-specific survival, and (E) overall survival for those undergoing BCS and mastectomy.

**Fig. 3:**
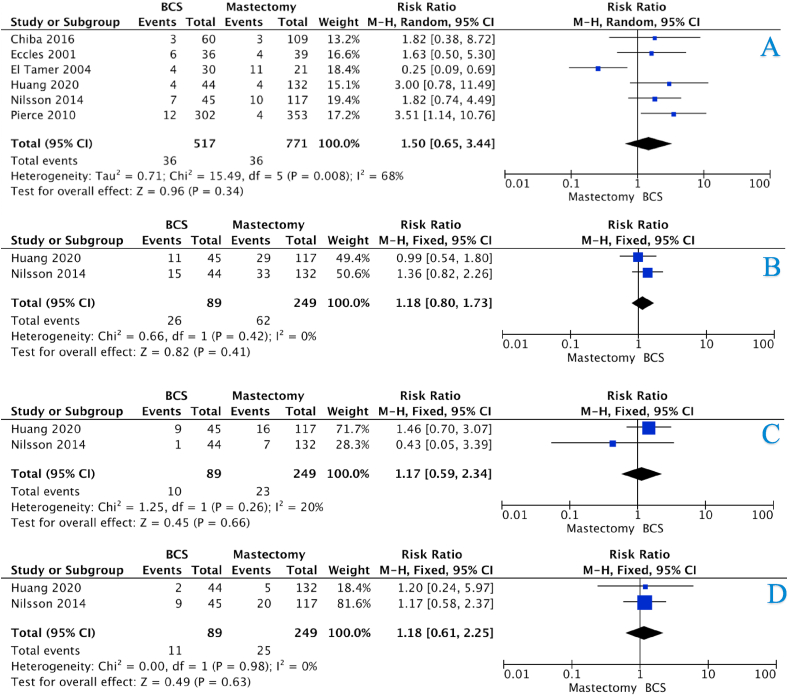
Forest plots illustrating 5-year (A) locoregional recurrence, (B) disease-free survival, (C) disease-specific survival, and (D) overall survival for those undergoing BCS and mastectomy.

**Fig. 4:**
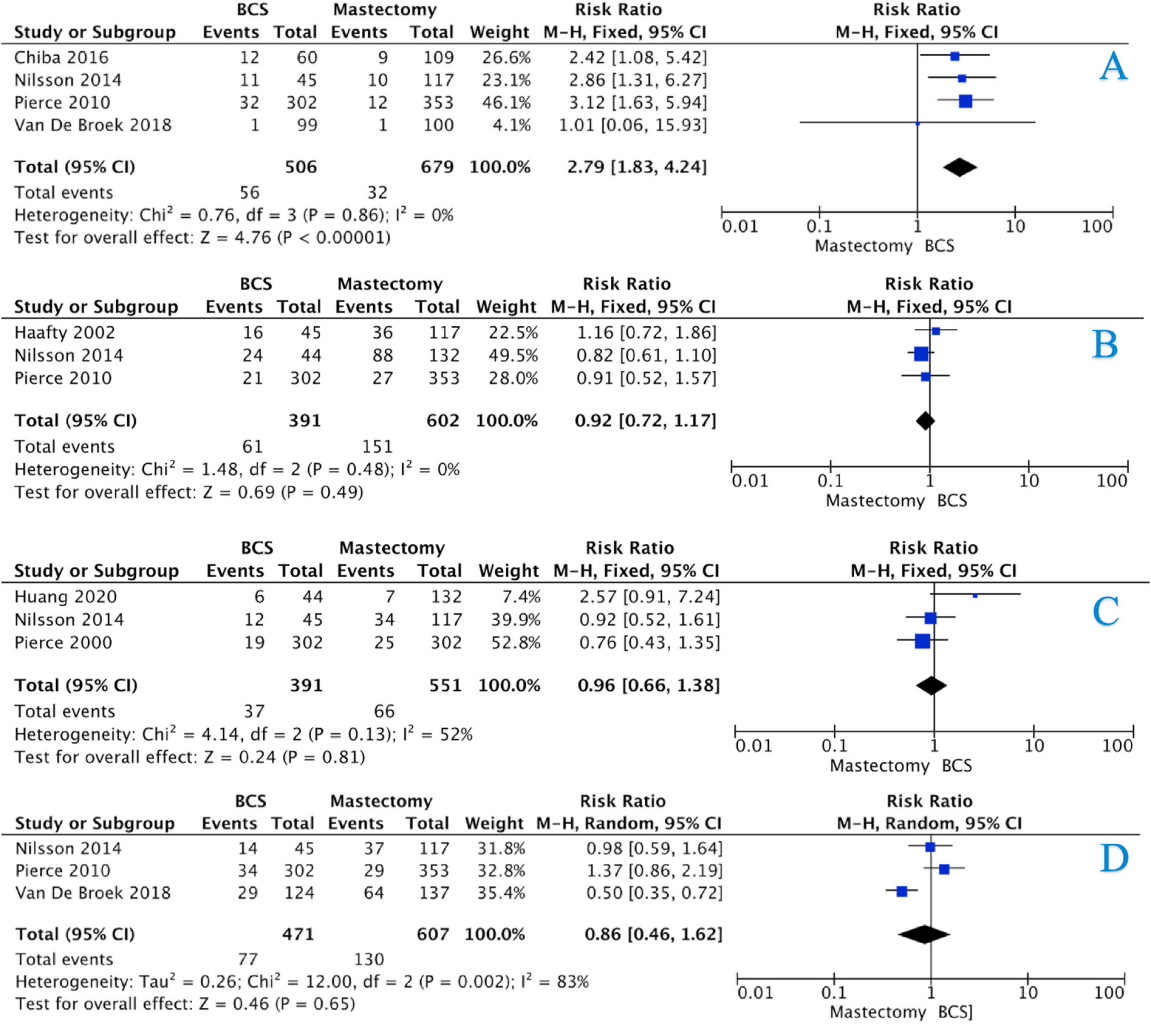
Forest plots illustrating 10-year (A) locoregional recurrence, (B) disease-free survival, (C) disease-specific survival, and (D) overall survival for those undergoing BCS and mastectomy.

**Fig. 5:**
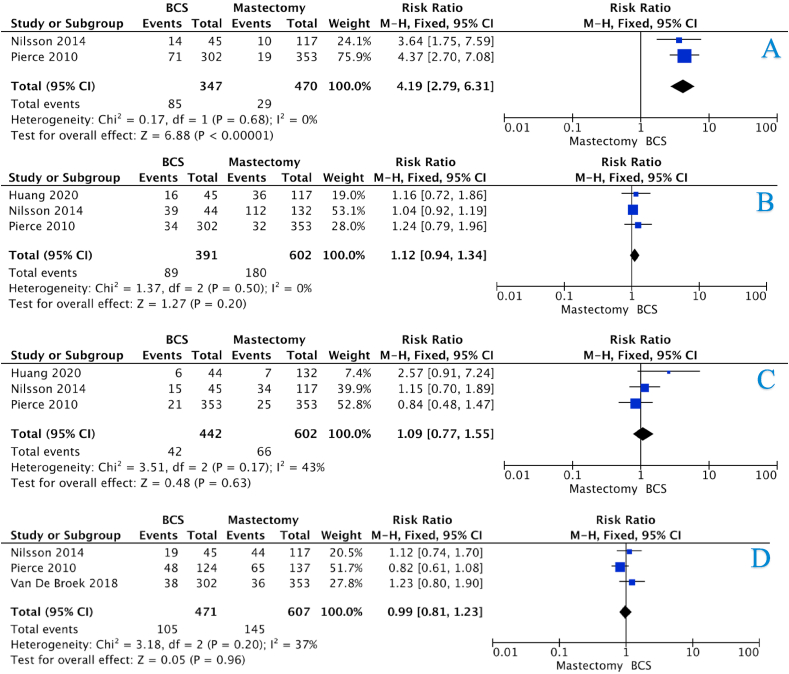
Forest plots illustrating 15-year (A) locoregional recurrence, (B) disease-free survival, (C) disease-specific survival, and (D) overall survival for those undergoing BCS and mastectomy.

The incidence of LRR for the BCS and mastectomy groups at 5-years were 14.7% *vs.* 4.8%, 15.5% *vs.* 4.7% at 10-years, and 27.5% *vs.* 6.2% at 15-years (all *P* < 0.001, †) (Table 4). There was an overall increased risk of LRR in those who underwent BCS (HR:4.54, 95%CI: 2.77–7.42, *P* < 0.001, heterogeneity (*I*^*2*^) = 0%) (Fig. 2A – 3 studies). The risk of LRR was equivocal in those who underwent BCS and mastectomy at 5-years (HR:1.50, 95%CI: 0.65–3.44, *P* = 0.420, *I*^*2*^ = 68%) (Fig. 3A – 6 studies). However, the risk of LRR increased at 10-years and 15-years respectively ([HR:2.79, 95%CI: 1.83–4.24, *P* < 0.001, *I*^*2*^ = 0%] & [HR:4.19, 95%CI: 2.79–6.31, *P* < 0.001, *I*^*2*^ = 0%]) (Fig. 4, Fig. 5A – 4 & 2 studies respectively).

**Table 4:** Recurrence and survival data for those undergoing breast conservation surgery and mastectomy at 5-years, 10-years and 15-years.

Parameter	BCS	Mastectomy	*P*-value
5-Year Recurrence and Survival			
LRR	262	36	<0.001^b^^a^
Free of recurrence	1519	735	
CLBC	74	N/R	N/R
Free of CLBC	552	N/R	
Recurrence	133	62	0.031^b^^a^
Free of recurrence	585	187	
DSS	85	23	0.036^b^^a^
Free of DSS	498	226	
Dead	101	27	<0.001^b^^a^
Alive	602	350	
10-Year Recurrence and Survival			
LRR	284	32	<0.001^b^^a^
Free of recurrence	1545	647	
CLBC	200	N/R	N/R
Free of CLBC	483	N/R	
Recurrence	241	151	0.881^a^
Free of recurrence	707	451	
DSS	144	66	0.002^b^^a^
Free of DSS	638	485	
Dead	309	135	0.141^a^
Alive	1160	600	
15-Year Recurrence and Survival			
LRR	310	29	<0.001^b^^a^
Free of recurrence	817	441	
CLBC	125	N/R	N/R
Free of CLBC	159	N/R	
Recurrence	90	180	0.016^b^^a^
Free of recurrence	302	422	
DSS	42	66	0.444^a^
Free of DSS	400	536	
Dead	111	145	0.507^a^
Alive	389	462	

LRR; locoregional recurrence, CLBC; contralateral breast cancer, DFS; disease-free survival, DSS; disease-specific survival, N/R; not reported Please note: Data from each study was not available to contribute to each outcome (e.g.: DFS, OS, etc.). Crude numbers are calculated from available data only.

a Denotes statistical significance.

b Fisher’s exact test.

### 3.6 Contralateral breast cancer

Overall, the incidence of contralateral breast cancer (CLBC) for those treated with BCS was 11.8% at 5-years, 29.3% at 10-years, and 45.6% at 15-years (Table 4). The risk of contralateral breast cancer development was equivalent for BCS or mastectomy (HR:1.51, 95%CI: 0.44–5.11, *P* = 0.510, *I*^*2*^ = 80%) (Figs. 3B–2 studies).

### 3.7 Disease-free survival

The incidence of disease recurrence for the BCS and mastectomy groups at 5-years were 15.5% *vs*. 24.9% (*P* = 0.031, †), 25.4% *vs.* 25.1% at 10-years (*P* = 0.881, †), and 23.0% *vs.* 29.9% at 15-years (*P* = 0.016, †) (Table 4). The risk of disease recurrence was equivocal for the BCS and mastectomy groups (HR:1.16, 95%CI: 0.78–1.72, *P* = 0.470, *I*^*2*^ = 44%) (Figs. 2C–3 studies), with no increased risk of disease recurrence after 5-years, 10-years or 15-years (Fig. 3, Fig. 4, Fig. 5B).

### 3.8 Disease-specific survival

The incidence of disease-specific recurrence for the BCS and mastectomy groups at 5-years were 14.3% *vs.* 9.2% (*P* = 0.036, †), 18.4% *vs.* 12.0% at 10-years (*P* = 0.002, †), and 9.5% *vs.* 11.0% at 15-years (*P* = 0.444, †) (Table 4). The risk of disease-specific recurrence was equivalent for the BCS and mastectomy groups (HR:1.58, 95%CI: 0.79–3.15, *P* = 0.200, *I*^*2*^ = 0%) (Fig. 2D– studies), with no increased risk of disease recurrence after 5-years, 10-years or 15-years (Fig. 3, Fig. 4, Fig. 5C).

### 3.9 Overall survival

The incidence of mortality for the BCS and mastectomy groups at 5-years were 14.4% *vs.* 9.2% (*P* < 0.001, †), 21.0% *vs.* 18.4% at 10-years (*P* = 0.141, †), and 22.2% *vs.* 23.9% at 15-years (*P* = 0.507, †) (Table 4). The risk of mortality was equivalent for the BCS and mastectomy groups (HR:1.10, 95%CI: 0.72–1.69, *P* = 0.660, *I*^*2*^ = 38%) (Fig. 2E– studies), with no increased risk of mortality after 5-years, 10-years or 15-years (Fig. 3, Fig. 4, Fig. 5D).

## 4 Discussion

In cases of sporadic breast cancer, benchmark practice recommends BCS as the “gold standard” procedure for tumour resection where feasible [40]. The evidence regarding BRCA mutation carriers with breast cancer is less certain. To our knowledge, this comprehensive systematic review and meta-analysis is the largest study evaluating the oncological safety profile of combined BCT versus mastectomy for BRCA mutation carriers following breast cancer diagnosis. The most important finding in this pooled analyses of over 3800 BRCA mutation carriers treated surgically with either BCS or mastectomy was the non-inferior survival analyses with respect to disease-free (DFS), disease-specific (DSS) and overall survival (OS) following up to a decade and a half of follow up. However, evidence of worse locoregional control following BCS overtime was also demonstrated, with a 4.5 times increased risk of LRR in the BCT group. This supports BCS as a valid option to be offered as a primary surgical strategy within BRCA mutation carriers, provided patients are appropriately counselled pre-operatively.

In patients with a family history, current international accepted practice for breast cancer management is to address the risk based on expression of key genes; in those with BRCA1/2 abnormalities, the current advice is to encourage bilateral mastectomy at diagnosis [41]. The data from this meta-analysis demonstrates BCS to have comparable oncological safety to mastectomy in BRCA mutation carriers with regards to survival. There was no survival disadvantage in a strategy encompassing BCT of the index case. Consequently, combined BCT should be discussed as a potential strategy allowing breast conservation following breast cancer diagnosis in BRCA mutation carriers, should the patient prefer such an approach. This is especially true where appropriate screening and surveillance is available as risks of LRR and new cancers are low at approximately 2.18% per year [42].

Oncological resection operates through zero-order kinetics, such that 100% of excised tumour cells are killed [43]. This concept remains true for both BCS and mastectomy, once surgeons successfully achieve clear margins in the setting of BCS. Although the inherent risk of mutation-related breast cancer occurring on the residual breast tissue spared by BCS remains, this analysis suggests this risk to be almost negligible when compared to outcomes following mastectomy. The authors wish to acknowledge that in certain clinical scenarios, such as those of increased tumour burden, diffuse in-situ disease, aggressive pathological characteristics, and in smaller breasts, mastectomy should remain the oncological procedure of choice. Moreover, counselling towards contralateral risk-reducing mastectomy should also be given to BRCA mutation carriers regardless of the patient’s initial choice regarding surgery for their cancer [44].

While seminal work by Fisher et al., demonstrated that long-term survival outcomes for sporadic breast cancer were non-inferior following combined BCT *vs.* mastectomy [6], this analysis demonstrates inferiority concerning LRR in BRCA patients treated with BCS, and an overt risk of LRR at intermediate- and long-term follow-up. These results are in agreement with a recent review by Co et al. [45] and refute any similar assertion concerning BCS for local control in BRCA mutation carriers. Eight of the included studies reported worse LRR outcomes following BCS relative to mastectomy, despite greater than 97% of patients receiving adjuvant radiotherapy. Valachis et al. previously indicated that combined BCT was initially equivocal in BRCA mutation carriers and sporadic breast cancer patients; however, the former were at an increased risk of LRR 7-years post resection [46], while Biglia et al. also highlighted increased LRR risk at greater than 15 years [47]. Observations from this study are congruent with both reports; and suggest that the clinical utility of mastectomy in achieving long-term locoregional control in BRCA carriers is explicit when compared to combined BCT. Increased LRR rates following BCS within BRCA carriers seems pertinent irrespective of adjuvant radiotherapy prescription, and the authors recommend patient counselling to be tailored as such.

An integral component of BRCA germline mutations is their pivotal role in driving oncogenesis within the younger patient - the median age of breast cancer diagnosis in this pooled analysis was only 41 years. Surgical decision making surrounding hereditary breast cancer must incorporate a myriad of personal, familial and cosmetic patient factors, the essence of which are of greater importance to the younger women [48]. These factors prove crucial in therapeutic and prophylactic surgical decision making. With almost half of the women included in this analyses being aged less than 40-years old, we wish to highlight the overall implication of the low risk of LRR at 10-years and 15-years following BCS. For these women, the incidence of LRR during this interval 5-to-15 years post resection is likely to occur within their 5th or 6th decade of life, long before index diagnoses of several other epithelial cancers including lung, colorectal and oesophageal carcinomas, all of which typically present in the 7th to 8th decades of life [[49], [50], [51]]. For the oncologist, relaying such implications to BRCA mutation carriers at primary diagnoses is of the utmost importance to ensure an accurate timeline of the potential clinical outcomes for the patient in the decades ahead.

### 4.1 Limitations

This systematic review and meta-analysis suffers from a number of inert limitations; studies included were limited to those published in the English language. Given the ethical implications surrounding BRCA testing and surgical best practice, there were no prospective studies for inclusion evaluating surgical outcomes in BRCA mutation carriers. Consequentially, all included studies were retrospective in nature indicating low-to-moderate levels of evidence. Moreover, patients included in this review underwent surgical resection during 8 decades, with patients recruited to their respective studies from 1959 to 2020; oncological practice has evolved in concordance with our understanding of the molecular properties of breast cancer in this period, which could limit the conclusions drawn from this analysis [52]. Overall, a limited number of studies met inclusion in our meta-analyses. Bilateral mastectomy has not been evaluated as a surgical option in cases of index cancers in this analysis. Difficulty differentiating possible data from studies published from two centres in the United States and the Netherlands may have affected outcomes, however ambiguity concerning this remains.

## 5 Conclusion

This study contradicts prevailing attitudes that suggest combined BCT confers worse survival when used as an approach to managing breast cancer in BRCA mutation carriers. While, combined BCT is associated with an increased risk of locoregional recurrence compared to mastectomy, irrespective of adjuvant radiotherapy prescription, these recurrences do not appear to adversely impact on survival when managed appropriately. This supports BCS as a valid option to be discussed as a primary surgical strategy allowing breast conservation for BRCA mutation carriers should the patient prefer such an approach. BRCA mutation carriers should be appropriately counselled regarding all surgical options following breast cancer diagnoses and this may allow them achieve the best outcome in an era of personalised medicine.
